# Expression of Baculovirus Anti-Apoptotic Genes *p35* and *op-iap* in Cotton (*Gossypium hirsutum* L.) Enhances Tolerance to Verticillium Wilt

**DOI:** 10.1371/journal.pone.0014218

**Published:** 2010-12-03

**Authors:** Juan Tian, Xueyan Zhang, Benguo Liang, Shanwei Li, Zhixia Wu, Qianhua Wang, Chunxu Leng, Jiangli Dong, Tao Wang

**Affiliations:** 1 State Key Laboratory of Agrobiotechnology, College of Biological Sciences, China Agricultural University, Beijing, China; 2 Key Laboratory of Cotton Genetic Improvement, Ministry of Agriculture, Institute of Cotton Research, Chinese Academy of Agricultural Sciences, Anyang, China; 3 Horticulture Department, Xinyang Agricultural College, Xinyang, China; 4 State Key Laboratory of Crop Biology, Shandong Agricultural University, Taian, China; Purdue University, United States of America

## Abstract

**Background:**

Programmed cell death plays an important role in mediating plant adaptive responses to the environment such as the invasion of pathogens. Verticillium wilt, caused by the necrotrophic pathogen *Verticillium dahliae*, is a serious vascular disease responsible for great economic losses to cotton, but the molecular mechanisms of verticillium disease and effective, safe methods of resistance to verticillium wilt remain unexplored.

**Methodology/Principal Findings:**

In this study, we introduced baculovirus apoptosis inhibitor genes *p35* and *op-iap* into the genome of cotton via *Agrobacterium*-mediated transformation and analyzed the response of transgenic plants to verticillium wilt. Results showed that *p35* and *op-iap* constructs were stably integrated into the cotton genome, expressed in the transgenic lines, and inherited through the T_3_ generation. The transgenic lines had significantly increased tolerance to verticillium wilt throughout the developmental stages. The disease index of T_1_–T_3_ generation was lower than 19, significantly (P<0.05) better than the negative control line z99668. After treatment with 250 mg/L VD-toxins for 36 hours, DNA from negative control leaves was fragmented, whereas fragmentation in the transgenic leaf DNA did not occur. The percentage of cell death in transgenic lines increased by 7.11% after 60 mg/L VD-toxin treatment, which was less than that of the negative control lines's 21.27%. This indicates that *p35* and *op-iap* gene expression partially protects cells from VD-toxin induced programmed cell death (PCD).

**Conclusion/Significance:**

*Verticillium dahliae* can trigger plant cells to die through induction of a PCD mechanism involved in pathogenesis. This paper provides a potential strategy for engineering broad-spectrum necrotrophic disease resistance in plants.

## Introduction

Verticillium wilt of cotton caused by *Verticillium dahliae* is one of the most serious diseases among cotton-producing regions worldwide. *Verticillium dahliae* is one subset of necrotrophic soilborne pathogens that form microsclerotia as surviving structures in the soil [1]. Once induced by root exudates, hyphae germinate and grow toward nearby roots, thought to be attracted by a nutrient gradient [2]. After penetrating the root cortex through wounds or by penetrating epidermal cells and crossing the endodermis, the fungus invades the vascular tissue. From there, it produces a large number of conidia and migrates through the xylem to the aerial parts of the plant, resulting in disease symptoms such as leaf vein browning and chlorosis, wilting, vascular discoloration, premature defoliation, and most severely, plant death [3], [4]. However, the molecular mechanisms involved in plant defense responses to verticillium wilt and the molecular mechanisms of verticillium diseases are poorly understood. The phenomenon whereby *V. dahliae* secretes toxins leading to verticillium wilt has received a great deal of attention. High-molecular-weight protein–lipopolysaccharide (PLP) complexes, glycoproteins, and cell wall-degrading enzymes were found to be present in the crude extracts containing verticillium toxin complexes [1]. Pegg [5] reported that *V. dahliae* produced phytotoxins and other molecules inducing host cell death. A glycopeptide toxin isolated from potato *V. dahliae* was associated with the production of disease symptoms in susceptible host plants[6]. Susceptible cotton cultivars are sensitive to toxin activity from crude verticillium extracts, resulting in ion leakage [7], [8]. Wang et al. [9] purified *V. dahliae* necrosis- and ethylene-inducing proteins (VdNEPs), which acted as elicitors in inducing phytoalexin production and programmed cell death in cotton suspension-cultured cells.

Programmed cell death (PCD) is an essential process not only for plant growth and development; it is also responsible for cell death in response to pathogen attacks and various abiotic stressors such as wounding, salt, cold, UV light, and herbicide (e.g., Paraquat) treatment [10], [11], [12]. Although the general understanding of plant PCD has progressed, its regulation and execution are still poorly understood, especially compared to the understanding of animal apoptosis. In animal cells, a core component of the PCD machinery is a family of cysteine-dependent, aspartate-specific proteases called “caspases.” During apoptosis, the initiator caspase is activated by pro-apoptotic signals and cleaves inactive pro-forms of effector caspases, thereby activating them. Effector caspases in turn cleave numerous cellular proteins, eventually leading to regulated apoptosis [13], [14]. The absence of caspase ortholog sequences in plants has been demonstrated by sequencing *Arabidopsis* and rice genomes [15]. However, previous reports have shown that animal caspase inhibitors can block plant PCD and most instances of plant PCD are associated with the induction of caspase-like events. Del Pozo and Lam [16] found that specific inhibitors of caspase-1 (Ac-YVAD-CMK) and caspase-3 (Ac-DEVD-CHO) have been observed to inhibit the occurrence of tobacco hypersensitive response (HR) induced by bacteria and *Tobacco mosaic virus* (TMV). Furthermore, they demonstrated caspase-1 and capase-3 participation in plant HR responses. The same caspase inhibitors also have been critically implicated in apoptosis of tobacco cells induced by isopentyladenosine and menadione [17], [18], [19].

Baculoviruses encode two mechanistically distinct apoptotic suppressors, inhibitor of apoptosis (IAP) and P35. Both viral proteins prevent premature insect cell death and thereby promote virus multiplication. The baculovirus anti-apoptotic proteins P35 and IAP are also effective in preventing plant PCD induced by bacterial, fungal, and viral infections [10], [19], [20], [21], [22], [23].

Baculovirus P35 was first isolated from *Autographa californica nuclear polyhedrosis virus* (AcMNPV) and is a broad-spectrum caspase inhibitor in nematode, *Drosophila*, and mammalian cells [24], [25], [26], [27]. The crystal structure of P35 is similar to a teapot. The “handle” of the teapot has a reactive site loop with a caspase cleavage site DQMD, which is recognized and cleaved by caspase. After cleavage, caspase and P35 form stable complexes between them via a covalent thioester bond, leading to caspase inactivity [28]. Derived from baculovirus *Orgyia pseudotsugata multicapsid polyhedrosis virus* (OpMNPV), Op-IAP is a potent inhibitor of apoptosis with two baculovirus IAP repeat (BIR) domains at the N-terminus and a really interesting new gene (RING)-finger domain at the C-terminus; it can inhibit the activity of caspases through the binding of its conserved BIR domains to the active sites of caspases *in vitro* and *in vivo* [29].

The transgenic maize embryogenic callus with baculovirus *p35* and *op-iap* inhibits *Agrobacterium tumefaciens*-induced PCD [19]. The transgenic tomatoes expressing *p35* can effectively inhibit AAL mycotoxin-induced cell apoptosis and reduce disease symptoms after inoculation with several necrotrophic tomato pathogens[20]. The narrow-leafed lupin containing the *p35* gene had significantly reduced necrotrophic disease symptoms [22].

Cotton is one of the most important cash crop in the world, the average annual cotton production loss as a result of Verticillium wilt is tremendous. In recent years, breeding new lines by genetic engineering to improve resistance to Verticillium wilt is one of important approaches for disease control. For example, transgenic coloured cotton plants over-expressing foreign *Gastrodia* anti-fungal protein were found to display robust resistance against *V. dahliae* in the field[30]. The crude leaf extracts from the transgenic cotton lines containing bean chitinase gene or *D4E1* gene (the synthetic antimicrobial peptide) inhibited the growth of *V. dahliae* in *vitro* [31], [32]. Transgenic cottons expressing glucose oxidase demonstrated significant increase in tolerance to *V. dahliae*, but vegetative growth, seed production and seedling vigour were also reduced[33]. In this study, we conducted the first attempt to introduce baculovirus apoptosis inhibitor genes *p35* and *op-iap* into the genome of cotton through *Agrobacterium*-mediated transformation and analyzed the response of transgenic plants to verticillium wilt. Results from this work reveal that caspase inhibitors can block plant cell death associated with cotton host–*V. dahliae* interactions. These results add to the growing evidence for pathogenesis-associated cell death in plants sharing common regulatory and mechanistic features with PCD in animals and evoke breeding strategies for verticillium wilt control.

## Materials and Methods

### Plasmid construction

The cDNA clones of baculovirus *p35* (900 bp) and *op-iap* (807 bp) in the insect expression vector pIE were a gift from Dr. Qianjun Li (University of Alabama at Birmingham, USA; GenBank Acc. Nos.: L22858 and L22564, respectively). PCR products were obtained using the following primers: *p35*, 5′-atgtgtgtaatttttccggtag-3′ and 5′-ttatttaattgtgtttaatatt -3′, and *op-iap*, 5′-atgagctcccgagcaattgg-3′ and 5′-ttacacttggtacatgcgcacc-3′, which were sequenced and subsequently cloned into pMD-18T (Takara Bio, Tokyo, Japan). The *p35* gene replaced the reporter gene of pBI121 through restriction with *BamH*I and *Sac*I enzymes to construct an intermediate vector pBI121*–p35*, with the *p35* gene under the 35S *Cauliflower mosaic virus* (CaMV) promoter and the nopaline synthase (nos) terminator. The GUS gene in the binary vector pCAMBIA 1301 (CAMBIA, Canberra, ACT, Australia) was replaced by the *op-iap* gene by restriction with *Bgl*II and *BstE*II, under the 35S CaMV promoter and the nos terminator, termed p1301*–iap*. The *p35* gene expression cassette obtained by *Pst*I and *EcoR*I restriction enzyme digestion was inserted into the p1301*–iap* vector of multiple cloning sites to construct the co-expression vector p1301*–iap–p35*.The plasmid was transformed into competent cells of *A. tumefaciens* strain EHA105 using the freeze–thaw method.

### Plant materials and plant transformation

Seeds of *Gossypium hirsutum* var. z99668 were provided by the Institute of Cotton Research of the Chinese Academy of Agricultural Sciences. Hypocotyl segments from 5–7-day-old sterile cotton seedlings were submerged for 10 minutes in EHA105 suspension cells which were grown to the late log phase (OD_600_ nm  = 0.6–0.8). The explants were blot-dried with sterile dry filter papers and subsequently transferred into cocultivation medium (MSB, which consisted of macro and microelements of MS medium, vitamins and amino acids of B5 medium, containing 0.1 mg/L 2,4-D, 0.1 mg/L KT, 0.1 mg/L IAA, 30 g/L glucose, 2 g/L Gelrite, pH 5.8) overlaid by a layer of filter paper for 48 hours at 26±2°C in the dark. After cocultivation, the explants were transferred to callus induction medium (MSB containing 0.1 mg/L 2,4-D, 0.1 mg/L KT, 0.1 mg/L IAA, 30 g/L glucose, 2 g/L Gelrite, pH 5.8) supplemented with 500 mg/L cefotaxime and 50 mg/L hygromycin. After 3–4 weeks, the resistant calli were transferred to differentiation medium (MSB containing 0.02 mg/L KT, 0.005 mg/L IAA, 30 g/L glucose, 2 g/L Gelrite, pH 5.8) to induce somatic embryogenesis. Embryoids were then grown on seedling medium (MSB containing 0.1 mg/L IBA, 30 g/L glucose, 2 g/L Gelrite, pH 5.8) to form regenerated plants. The regenerated plants from independent resistant calluses were grafted onto the rootstock seedlings[34] and then moved to greenhouse cultivation. Hygromycin at 80 mg/L was used to paint leaves of the regenerated plants to evaluate hygromycin resistance. Leaves of susceptible seedlings will change color from green to yellow after 5–7 days. After 3 repeated applications, hygromycin-resistant plants were kept for further molecular analysis and disease essay. After self-pollination, T_1_ generation seeds were collected, and self-pollinated T_2_ and T_3_ generation seeds were harvested over the next 2 years, respectively.

### Expression analysis of transgenes

To determine the integration of cassettes in the genome, polymerase chain reaction (PCR) was conducted using specific primer pairs (*p35*: 5′-cccacagatggttagagagg-3′ and 5′-gtagtagtcgttgcgttcgt-3′ and *op-iap*: 5′-atgagctcccgagcaatt-3′ and 5′-acgcctcagtcatcaccc -3′) to amplify the CaMV 35S promoter *p35* region and *op-iap* from T_1_ to T_3_ transgenic cotton plants. Genomic DNA (gDNA) was extracted and purified from young leaves using the method described by Paterson et al. [35]and Li et al. [36]. PCR of the *p35* gene was conducted in a Touchgen thermal cycler (Techne, Cambridge, UK) using the following program: initial denaturing at 95°C for 5 minutes followed by 35 cycles of denaturing at 95°C for 50 seconds, annealing at 55°C for 50 seconds, extension at 72°C for 1 minute 30 seconds, and final extension at 72°C for 10 minutes. For the *op-iap* gene, the same program was used except for the annealing step, which was 58°C for 50 seconds, extension at 72°C for 1 minute. PCR products obtained were analyzed by 1% agarose gel electrophoresis followed by ethidium bromide staining.

For Southern blot analysis, 30 µg gDNA of T_3_ transgenic cotton plants and non-transformed negative controls was digested with *EcoR*I. The restriction fragments were size-fractionated by 0.8% (w/v) agarose gel electrophoresis and transferred to a Hybond-N+ nylon membrane (Amersham Pharmacia Biotech, UK). Probes were prepared from purified PCR products of the *p35* (870 bp) and *op-iap* (583 bp) coding region. The labeling of probe, prehybridization, hybridization and detection were performed by the protocol of DIG High Prime DNA Labeling and Detection Starter KitI (catalog no. 11745832910; Roche Applied Science, Mannheim, Germany).

The transcription of *p35* and *op-iap* was detected by semi-quantitative reverse transcription (RT)-PCR. RNA was extracted from young leaves of cotton following Li et al. [36] and all RNA samples were treated with DNaseI (Takara Bio) to prevent contamination with gDNA. One µg of the treated RNA was reverse-transcribed into cDNA using M-MLV Reverse Transcriptase following the manufacturer's protocol (Takara code: D2639A). PCR of 1 µl first-strand cDNA was used to amplify genes by using specific primer pairs (*p35*:5′-gtagaaatcgacgtgtcccagac -3′ and 5′-tgagcaaacggcacaataactt -3′ ; *op-iap*: 5′-atgagctcccgagcaatt-3′ and 5′-acgcctcagtcatcaccc -3′) and the following programs: 95°C for 5 min, 28 cycles of denaturing at 95°C for 40 seconds, annealing at 49°C (*p35*) or 58°C (*op-iap*) for 40 seconds, extension at 72°C for 40 seconds and final extension at 72°C for 10 minutes. The RT-PCR for the cotton house-keeping gene *histone-3* (GenBank Acc. Nos.: AF024716) was performed with specific primer (5′-ataccgtcctggaactgttgctc-3′ and 5′-aacatatcagacgccccacttca-3′) under the same conditions as described above to determine whether the equal amounts of total RNA were used in the RT-PCR reactions among samples. All RT-PCR expression assays were independently performed and analyzed three times under identical conditions.

Soluble proteins were extracted with 0.5 ml of an extraction buffer [23]. Protein concentration was measured according to the method of Bradford [37] using a Bio-Rad reagent (Bio-Rad, Hercules, CA) with bovine serum albumin (BSA; Sigma, St. Louis, MO) as a standard. After boiling for 10 minutes in the sample buffer, 10 µl of proteins was loaded in a 12% SDS-polyacrylamide gel, and the proteins were subsequently transferred onto a Hybond-N membrane (Bio-Rad) via a semidry trans-electroblotter. After blocking for 1 hour in TBST buffer [20 mM Tris (pH 7.5), 150 mM NaCl, and 0.1% Tween 20] with 5% nonfat dry milk at room temperature, the membrane was probed with the anti-*p35* (1∶1500, catalog no. IMG-5740; IMGENEX, San Diego, CA) and the anti-*iap* antibodies (1∶1000, catalog no. GTX23930; GeneTex, Irvine, CA). Goat anti-rabbit IgG alkaline phosphatase conjugate (1∶1000, catalog no. bsap-0295G; Beijing Boaosen Biotechnology, Ltd., Beijing, China) was used as a secondary antibody and the hybridization membrane was washed three times using NBT/BCIP chromogenic stain.

### Pathogen cultivation and inoculation

The techniques used for disease assessment in the field were those previously reported by Wang et al. [30].The transgenic cotton lines and non-transformed negative controls with three replications were grown in the cotton verticillium wilt artificial nursery at the Institute of Cotton Research of the Chinese Academy of Agricultural Sciences. *Verticillium dahliae* with moderate virulence can cause the susceptible control (ji11) infection rate of 80%, or a disease index above 50. Disease severity was surveyed respectively in the seedling stage, flowering stage, and boll stage. According to the standard protocol developed by Wang et al. [30], the degree of infection by pathogenic fungi was divided into five grades with disease scores ranging between 0 and 4 (0: healthy plants, no fungal infection; 1: <25% of the leaves showing yellowing or abnormal yellow spots; 2: ≤25 to <50% of the leaves showing yellow spots and curled leaf edges; 3: ≤50 to <75% of the leaves showing brown spots and curled leaf edges with some leaves dropping; and 4: ≥75% of the leaves produce yellow or yellow irregular spots between the main vein of leaves). The infection rate and disease index were calculated according to the following formula:

infection rate (%)  =  (the number of total infected plants/the number of total checked plants) ×100

disease index  =  [(Σ disease scores × number of infected)/total checked plants × highest grade disease (4)] ×100.

The mean and standard deviation (SD) were calculated based on three replications. The statistical significance (P<0.05) between the wild-type plant and each transgenic lines was tested through student's t-test using Statistical Analysis System (SAS) (SAS Institute Inc., USA).

### Preparation of crude VD-toxin from *V. dahliae*

*Verticillium dahliae* Kleb (V229), a strong pathogenic and non-defoliating strain originally isolated from diseased cotton tissue and obtained from Prof. Yingzhang Li (College of Biological Sciences, China Agricultural University), was first activated in potato dextrose agar medium at 22°C in the dark for 5 days. The fungal mycelia were cultured in Czapek's medium (containing 30.0 g sucrose, 3.0 g NaNO_3_, 1.0 g K_2_HPO_4_, 1.0 g MgSO_4_·7H_2_O, 1 g KCl, and 0.01 g FeSO_4_·7H_2_O in 1 L of distilled water)[7] by shaking at 130 rpm and 28°C for 14 days. The cultures were centrifuged at 10,000× *g* for 30 minutes to remove spores and the supernatant was filtered with two layers of filter paper. After lyophilization, the freeze-dried filtrate was then dissolved in distilled water; the solution was filtered through a 0.45-µm filter (Millipore, Billerica, MA) and used as a crude VD-toxin extract [38], [39], [40]. Protein content in crude extracts was determined according to Bradford [37] with BSA (Sigma) as a standard.

### Nuclear DNA fragmentation

The leaves from 6-week-old transgenic lines (KB1) and non-transformed negative control seedlings were treated with 250 mg/L VD-toxin for 24 and 36 hours, then frozen in liquid nitrogen and ground into a fine powder. DNA was isolated as described by Paterson et al. [35]and Li et al. [36]. Four µg DNA treated with 25 µg/ml DNase-free RNase A (Takara) for 1 hour at 37°C electrophoreses on a 2% agarose gel. After electrophoresis, the gel was stained with 0.5 µg/ml ethidium bromide for 30 minutes, de-stained in 1 mM MgSO_4_ for 1 hour, and observed and photographed on a UV light box [41], [42].

### Flow cytometry analysis of cell death

Cotton protoplasts were isolated from the transgenic lines (KB1) and non-transformed negative controls. The washed 4-week-old leaves were briefly dried and cut into small leaf strips (0.5 mm) with a razor blade; then the strips were incubated in an enzyme solution [3% (w/v) cellulose R-10 (Yakult Honsha, Tokyo, Japan), 1.5% Macerozyme R-10 (Yakult Honsha), 0.4 M mannitol, 20 mM MES, 10 mM CaCl_2_, 0.2 mM KH_2_PO_4_, 1 mM KNO_3_, 1 mM MgSO_4_, 0.1 µM CuSO_4_, 10 µM KI, pH 5.8] in the dark by gentle shaking. The protoplasts were separated from undigested materials using 75-µm stainless steel mesh and the crude protoplast filtrates were sedimented by centrifugation for 2 minutes at 700 rpm. Finally, the purified protoplasts were resuspended in W5 solution (154 mM NaCl, 125 mM CaCl_2_, 5 mM KCl, 5 mM glucose, 1.5 mM MES-KOH, pH 5.8) [43]. Protoplasts were counted using a hemocytometer.

Cotton protoplasts were treated with 30 mg/L and 60 mg/L VD-toxin for 3 hours and then the number of dead protoplastes was determined using a propidium iodide (PI) fluorescent probe. Loss of plasma membrane integrity around apoptotic cells can be demonstrated using fluorogenic compounds such as PI, which is excluded by an intact membrane. Once the cell membrane permeability increases, PI enters the cell and binds stoichiometrically to nucleic acids. Fluorescence emission is proportional to the degree of cell death. Protoplasts were incubated with 30 µg/ml PI in culture medium for 5–10 minutes [44] and PI staining was quantified in the FL-2 channel of a FACSCalibur instrument (Becton, Dickinson and Company, Franklin Lakes, NJ) using the CELL Quest program (Becton, Dickinson and Company). Each sample for flow cytometry analysis contained 10,000 protoplasts.

## Results

### Cotton transformation

**Figure 1:**
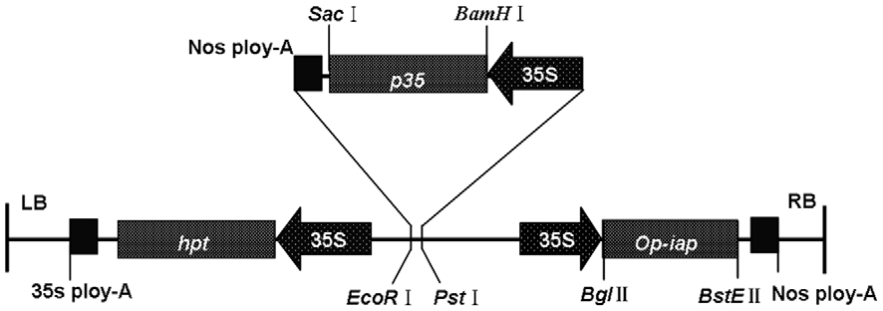
Schematic map of the T-DNA region of *p35* and *op-iap*. The *p35* gene replaced the reporter gene of pBI121 through restriction with *Bam*HI and *Sac*I enzymes to construct an intermediate vector pBI121*–p35*. The *op-iap* gene replaced the reporter gene of pCAMBIA 1301 by restriction with *Bgl*II and *BstE*II termed p1301*–iap*. The *p35* gene expression cassette obtained by *Pst* I and *EcoR* I restriction enzyme digestion was inserted into p1301*-iap* of the multiple cloning sites to construct the co-expression vector p1301-*iap*-*p35*.

The transformation binary vector carrying an anti-apoptotic baculovirus *p35* gene cassette (CaMV 35S promoter-*p35* ORF-Nos terminator) and the *op-iap* gene cassette (CaMV 35S promoter-op-iap ORF-Nos terminator) was designated p1301–*p35*-*iap* (Fig. 1). *Agrobacterium*-mediated transformation of *G. hirsutum* var. Z99668 using the co-expression vector p1301–*p35*–*iap* (Fig. 1) yielded 19 independent T_0_ transformation events after several rounds of selection. Putative transformed shoots from 17 transformant events were successfully rooted by grafting methods and matured. All 17 putative transgenic cotton plants had similar phenotypes as non-transformed negative controls with respect to growth, leaf shape, and flowering. After 3 years of self-pollination, T_1_, T_2_ and T_3_ seeds were harvested. All offspring seeds had comparable sizes and appearances to the non-transformed negative controls (F_1_, F_2_ and F_3_). The analyses of three putative transformed plants labeled KB1, KB2, and KB3, which were randomly chosen from 17 putative transgenic cotton plants, are provided in this report.

### Integration and expression of *p35* and *op-iap* in transgenic lines

**Figure 2:**
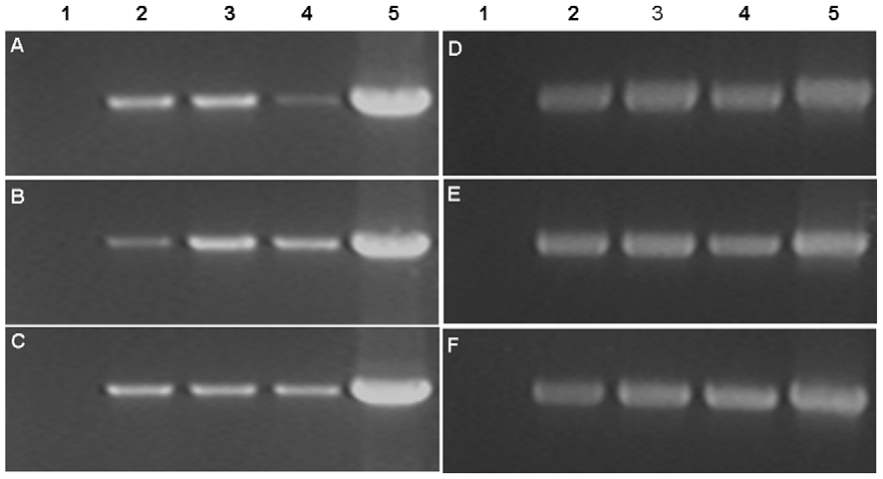
PCR analysis of *p35* and *op-iap* genes in T_1_-T_3_ generations of the transgenic cotton lines. PCR products from genomic DNA of the three transgenic T_1_–T_3_ generation and non-transformed negative control plants. (A) the presence of *p35* in T_1_ generation. (B) the presence of *p35* in T_2_ generation. (C) the presence of *p35* in T_3_ generation. (D) the presence of *op-iap* in T_1_ generation. (E) the presence of *op-iap* in T_2_ generation. (F) the presence *op-iap* in T_3_ generation. Lane 1 the negative control plant; lanes 2, 3, 4 the three transgenics lines (KB1, KB2, KB3); lane 5 the p1301-*p35*-*iap* co-expression vector control.

**Figure 3:**
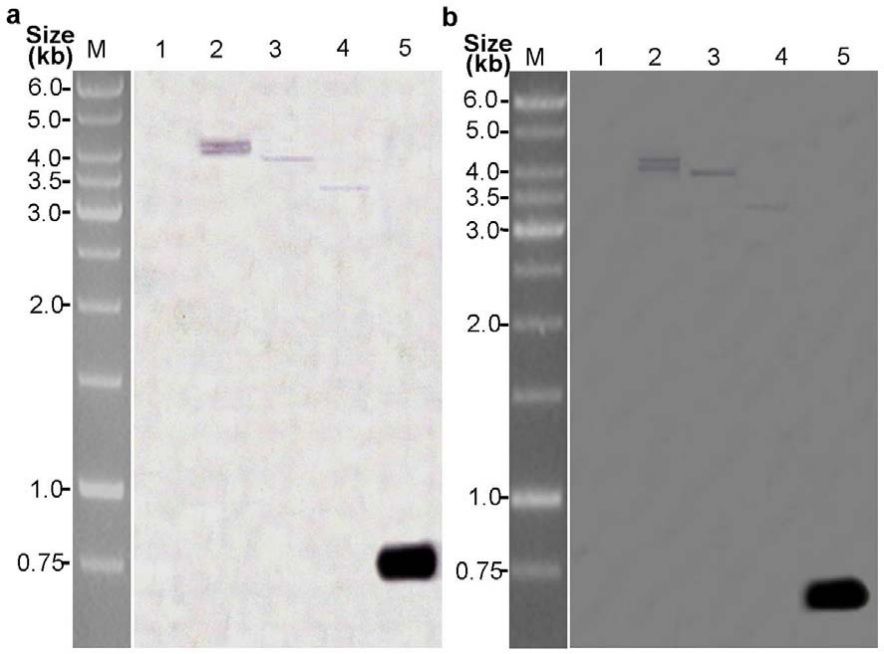
Southern blot analysis of three T_3_ transgenic cotton lines. Southern blot hybridization analysis of genomic DNA extracted from leaves of three transgenic cotton plants and non-transformed negative control plants. (a) DNA from transgenic cotton plants and non-transformed negative control plants was digested with *EcoR*I and hybridized with a DIG-labeled *p35* probe (870 bp); (b) DNA from three transgenic cotton plants and non-transformed negative control plants was digested with *EcoR*I and hybridized with a DIG-labeled *op-iap* probe (583 bp); Lane 1 the negative control plant; lanes 2, 3, 4 the three transgenics lines (KB1, KB2, KB3); lane 5: positive control [purified PCR products of the *p35* (870 bp) and *op-iap* (583 bp) coding region]; lane M molecular mass marker is GeneRuler TM 1 kb DNA ladder (MBI Fermentas, Maryland, USA).

The integration of two cassettes into the genomes of the three transgenic plants for each generation was confirmed by PCR and southern blot using genomic DNA of the T_3_ transgenic plants. Using a primer internal to the CaMV 35S promoter and to *p35*, and a primer internal to the *op-iap* gene, PCR products of the expected sizes of 1266 bp (CaMV 35S-*p35*) and 583 bp (*op-iap*) were obtained from all three T_1_–T_3_ transgenic samples for each transgenic plant (Fig. 2). The product sizes were identical to amplification from the p1301–*p35*–*iap* co-expression vector, whereas no PCR products were obtained from the amplification of genomic DNA in non-transformed negative controls. The DNA of three transgenic T_3_ plants and the untransformed control plant was digested with *EcoR* I, and respectively hybridized with DIG-labeled *p35* and *op-iap* gene probes. All three transgenic plants exhibited hybridization signals with two probes while the non-transformed control did not. With *p35* probe, plant KB1 showed two hybridization signals, while KB2 and KB3 had one band of hybridization respectively (Fig. 3a). The hybridization signals with *op-iap* probe displayed the same patterns and the copy numbers as *p35* (Fig. 3b). The results of this analyses indicated the presence and stable integration of the *p35* and *op-iap* genes into the genome of all three transgenic plants. PCR analysis of the progeny of these transgenic lines of cotton suggested that *p35* and *op-iap* were able to be stably inherited into the progeny.

**Figure 4:**
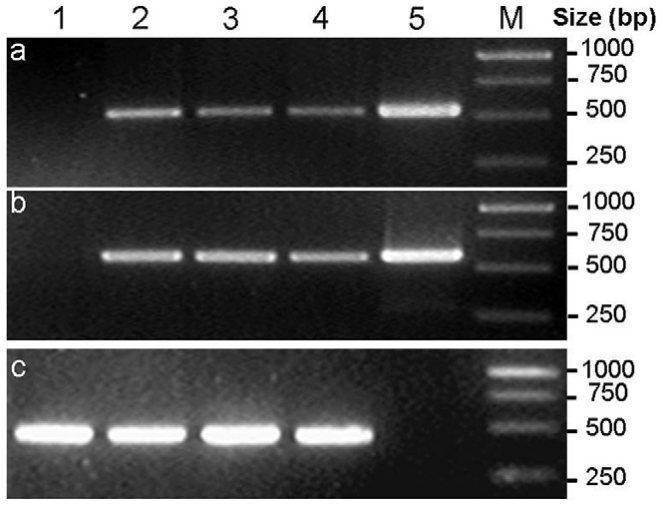
Semi-quantitative RT-PCR analysis of *p35* and *op-iap* expression in three T_3_ transgenic cotton lines. Semi-quantitative RT-PCR products from RNA extracted from the young leaves of three T_3_ transgenic plants and non-transformed negative controls. (a) *p35* expression. (b) *op-iap* expression. (c) house-keeping gene *histone-3* expression. Lane 1 the negative control plant; lanes 2, 3, 4 the three T_3_ transgenic lines (KB1, KB2, KB3); lane 5 the p1301-*p35*-*iap* co-expression vector control; lane M molecular mass marker is GeneRuler ^TM^ 1 kb DNA ladder (MBI Fermentas, Maryland, USA).

To detect accumulation of *p35* and *op-iap* transcripts, semi-quantitative RT-PCR of isolated total RNA from the three T_3_ transgenic plants and the non-transformed negative controls was performed to demonstrate expression of the *p35* and *op-iap* genes. PCR amplification of cDNA from all three T_3_ transgenic plants and the plasmid control template yielded the expected products of 533 bp or 583 bp in length, respectively, while no corresponding products were amplified from non-transformed negative controls (Fig. 4). DNA sequence analysis showed that the DNA sequences integrated in the three T_3_ transgenic plants were identical to that of the region amplified from the p1301–*p35*–*iap* co-expression vector control.

**Figure 5:**
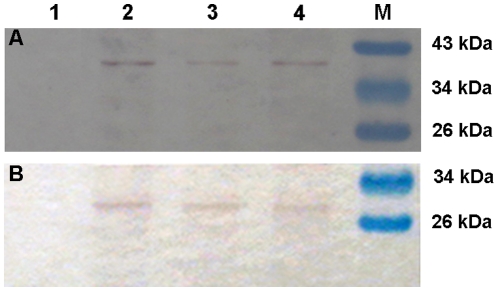
Western blot assays of expression of P35 and OP-IAP protein in T_3_ transgenic cotton lines. The total soluble protein extracted from the young leaves of the T_3_ transgenic lines and non-transformed negative controls. (A) P35 expression. (B) OP-IAP expression. Lane 1 the negative control plant; lane 2, 3, 4 the three T_3_ transgenic lines (KB1, KB2, KB3); lane M molecular mass marker is PageRuler ^TM^ prestained protein ladder (MBI Fermentas, Maryland, USA).

To further confirm the protein expression of *p35* and *op-iap* in transgenic plants, total soluble protein was extracted from the three T_3_ transgenic plants and non-transformed negative controls and analyzed by Western blotting. Figure 5 shows that using anti-*p35* and anti-*iap* antibodies revealed the presence of 35-kDa and 30-kDa protein bands, which are the same size as P35 and OP-IAP reported by Birnbaum et al. [45]and Wang et al. [23] in transgenic cotton lines KB1, KB2, and KB3. The non-transformed negative controls showed no positive signal for P35 and IAP proteins.

### Disease resistance survey of the T_1_, T_2_, and T_3_ generations

**Figure 6:**
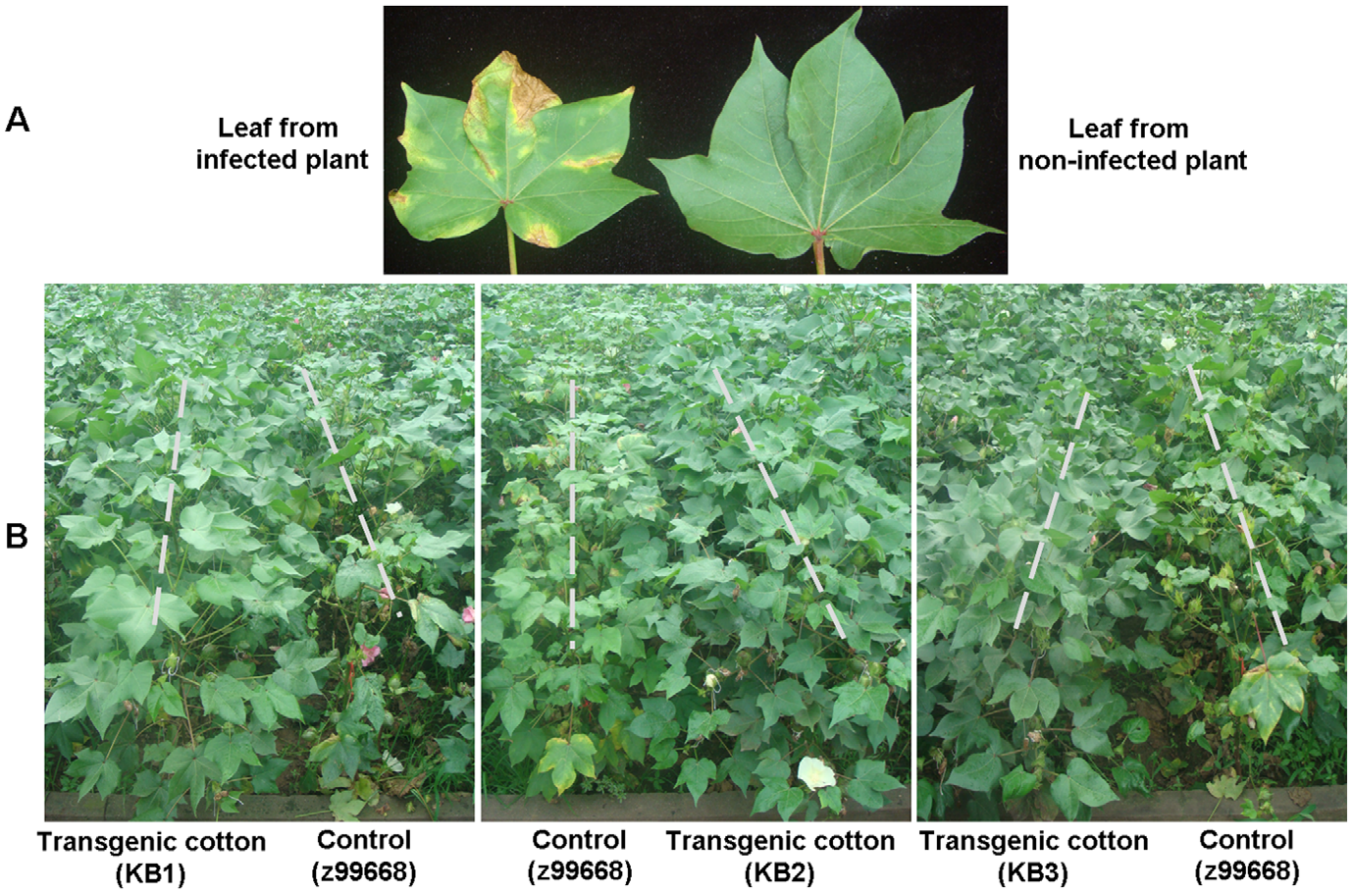
The disease resistance phenotype of T_3_ transgenic cotton lines in the verticillium wilt artificial nursery. A. Disease symptoms of verticillium wilt at the cotton flowering stage. The leaf shows yellow or brown spots and curled leaf edges. B. The disease resistance phenotype of the T_3_ transgenic cotton lines (KB1, KB2, KB3) and the negative control lines (z99668) at the flowering stage in the cotton verticillium wilt artificial nursery. Three T_3_ transgenic cotton lines did not exhibit disease symptoms, while the leaves of the negative control lines (z99668) showed yellow or brown spots and curled leaf edges, some leaves dropped.

To accurately evaluate disease resistance in transgenic cottons, we used an artificial nursery to evaluate resistance to verticillium wilt of cotton. Disease severity was expressed as a disease index (DI). Analysis of verticillium wilt resistance or susceptibility of the transgenic cotton lines was based on visual assessment of chlorosis and necrosis symptoms of leaves. The infection rate and disease index were calculated for each plant using the formula stated earlier. Disease evaluation showed that the disease index of T_1_, T_2_, and T_3_ generations of the three transgenic cotton lines (KB1, KB2, and KB3) throughout the developmental stages were lower than 19 and the resistance levels were between highly resistance (HR) to disease and disease-resistant (R), which was significantly (P<0.05) better than those of the negative control line z99668 (Table 1, Fig. 6). The results of the pathogenicity assay were consistent with that of the molecular analyses, which demonstrated that the strong resistance of the KB1, KB2, and KB3 lines to verticillium wilt was due to the heterologous expression of *p35* and *op-iap*.

**Table 1:** Analyses of disease resistance of transgenic cotton lines.

	plant number	Infectionrate(%)			DiseaseIndex(DI)			Disease resistant(R)/Tolerance(T)/Susceptible(S)		
Transplant		T_1_/F_1_	T_2_/F_2_	T_3_/F_3_	T_1_/F_1_	T_2_/F_2_	T_3_/F_3_	T_1_/F_1_	T_2_/F_2_	T_3_/F_3_
	KB1	16.7^*^±2.3	54.2^*^±0.96	38.1^*^±1.49	14.6^*^±1.05	17.9^*^±0.87	13.5^*^±0.45	R	R	R
	KB2	24.1^*^±0.75	47.3^*^±1.04	44.1^*^±0.82	16.9^*^±0.45	15.1^*^±0.89	18.8^*^±0.56	R	R	R
	KB3	10.7^*^±1.32	53.6^*^±0.62	39^*^±0.66	8.8^*^±0.66	17.1^*^±0.46	15.9^*^±0.56	HR	R	R
Control	Z99668	35.8±0.85	49.6±0.7	56.4±0.95	33.8±0.66	20.6±0.7	25.5±0.95	T	T	T

Disease evaluation standards: HR, highly resistant plants with a DI value between 0 and 10.0; R, resistant plants with a DI value between 10.1 and 20.0; T, disease tolerance plants with a DI value between 20.1 and 35.0; S, susceptible plants with a DI value >35.0. The means standard deviations (SD) were calculated based on three replications. Asterisks indicate statistical significance (P<0.05) between the control plant(z99668) and each transgenic lines (KB1, KB2 and KB3).

### DNA laddering in fungus-infected plants

**Figure 7:**
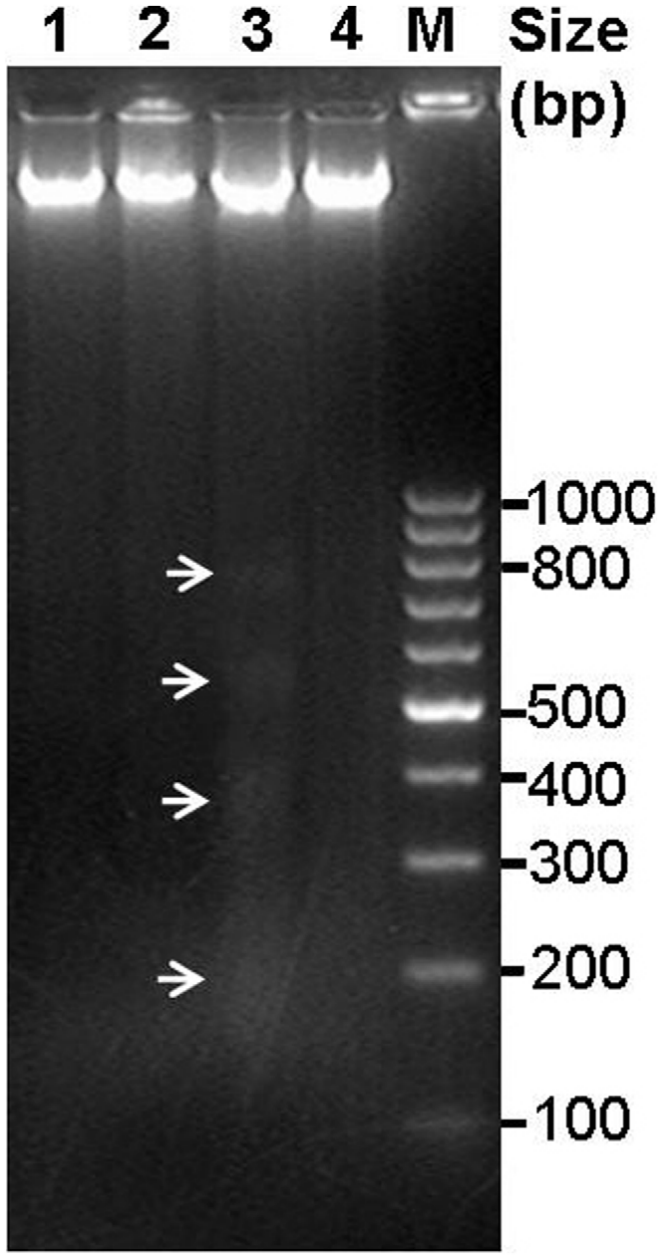
DNA laddering in cotton lines after treatment with VD-toxins. Ethidium bromide-stained agarose gel (2%) of DNA extracted from T_3_ transgenic (KB1) leaves after treatment with 250 mg/L VD-toxin for 24 hours (lane 2) and 36 hours (lane 4), DNA extracted from the negative control leaves after treatment with 250 mg/L VD-toxin for 24 hours (lane 1) and 36 hours (lane 3). lane M molecular mass marker is GeneRuler ^TM^ 100 bp DNA ladder (MBI Fermentas, Maryland, USA).

To test whether VD-toxins induce PCD in cotton, genomic DNA was isolated from T_3_ transgenic (KB1) and non-transformed negative control leaves after being treated with 250 mg/L VD-toxin for 24 and 36 hours. DNA ladders are the hallmark feature of PCD, as a result of the activation of cell death-specific endonucleases such as DNases that cleave the DNA at the linker regions between oligonucleosomes to produce nucleosomal and oligonucleosomal DNA fragments (180 bp and multiples of 180 bp) that generate a characteristic “ladder” pattern during agarose gel electrophoresis. As shown in Fig. 7 (lane 3), the isolated DNA from non-transformed negative control leaves after treatment with 250 mg/L VD-toxin for 36 hours was fragmented and formed characteristic ladders that differed by just under 200 bp, whereas the transgenic line treated with 250 mg/L VD-toxin for 36 hours had no such ladder formation (Fig. 7, lane 4). That VD-toxins trigger plant cells to die by induction of a PCD mechanism involved in pathogenesis and that the expression of *p35* and *op-iap* genes can inhibit such mechanism seem reasonable.

### Flow cytometry analysis of cell death

**Figure 8:**
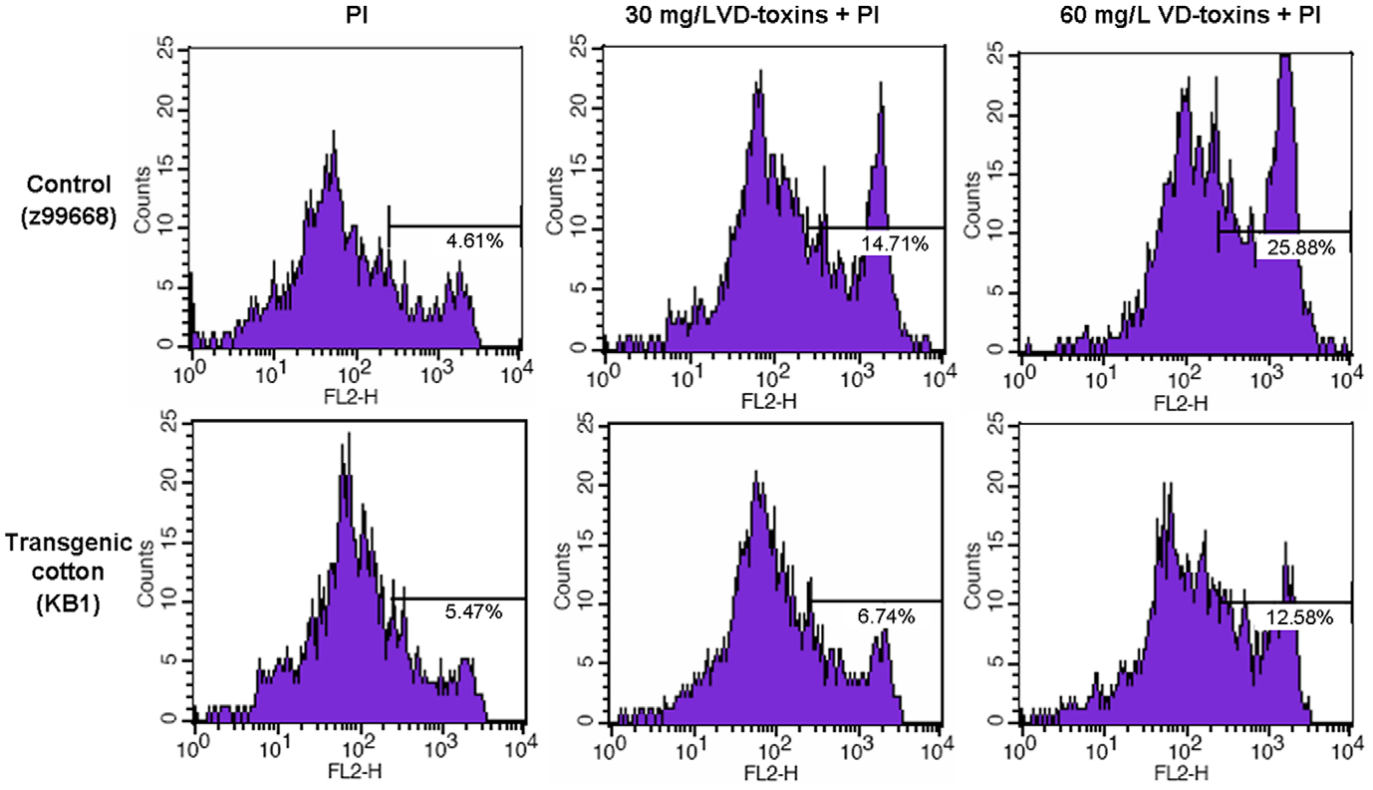
Flow Cytometry Analysis of Cell Death Induced by VD-toxins. The protoplasts isolated from the T_3_ transgenic lines (KB1) and non-transformed negative controls were treated with 30 mg/L and 60 mg/L VD-toxin for 3 hours and analyzed by flow cytometry after PI staining. Flow cytometry analysis was conducted using 10,000 protoplasts per sample. Percentages of dead protoplasts are indicated. Vertical axis refers to PI fluorescence intensity and the abscissa refers to the number of protoplast. Data are representative of three independent experiments.

To determine whether *p35* and *op-iap* gene expression protects cells from VD-toxin-induced PCD, protoplasts isolated from the T_3_ transgenic lines (KB1) and non-transformed negative controls were treated with 30 mg/L and 60 mg/L VD-toxin for 3 hours to induce cell death and then analyzed by flow cytometry after PI staining. After VD-toxin treatment, the percentage of protoplast death for the transgenic lines (KB1) and non-transformed negative controls all displayed an increase, but the percentage of dead protoplasts for non-transformed negative controls had a significantly higher increase than the transgenic lines (KB1). As shown in Fig. 8, in the 30 mg/L VD-toxin treatment, the percentage of dead protoplasts for the non-transformed negative controls and the transgenic lines (KB1) increased by 10.1% and 1.27%, respectively. In the 60 mg/L VD-toxin treatment, the percentage of dead protoplasts increased by 21.27% and 7.11%, respectively. These results indicate that *p35* and *op-iap* gene expression partially protects cells from VD-toxin-induced PCD.

## Discussion

PCD plays an important role in mediating plant adaptive responses to several stimuli, such as senescence, or in response to pathogens and environmental stresses. In this study, we introduced anti-apoptotic *p35* and *op-iap* genes from baculovirus into the genome of cotton and analyzed the response of transgenic plants to verticillium wilt. The results showed that the construct containing *p35* and *op-iap* was not only stably integrated into the cotton genome and expressed in the transgenic lines, but also able to be inherited through to the T_3_ generation. Three transgenic lines (KB1, KB2, and KB3) performed markedly (P<0.05) better than the negative control line z99668 when inoculated with *V. dahliae* in the verticillium wilt nursery room. After treatment with 250 mg/L VD-toxins for 36 hours, DNA from the non-transformed negative control leaves was fragmented, whereas that from the transgenic lines was not. The percentage of dead protoplasts from the non-transformed negative controls was higher than that from the transgenic cotton lines after VD-toxin treatment, indicating that *p35* and *op-iap* gene expression in transgenic lines enhances the tolerance to verticillium wilt and partially protects cells from VD-toxin-induced cell death.

Previous studies have reported results consistent with our observations. Hansen [19] showed that transgenic maize embryogenic callus with the baculovirus apoptosis inhibitory genes *p35* and *op-iap* can inhibit *Agrobacterium*-induced PCD. Tobacco plants expressing the human anti-apoptotic genes *bcl-2*, *bcl-xL*, nematode *Ced-9*, and baculovirus *Op-iap* showed increased resistance to the tomato spotted wilt virus (TSWV), with fewer local lesions and prevention of systemic spread of the virus [10]. Lincoln et al. [20]and Wang et al. [23]showed that expression of the baculovirus *p35* gene effectively blocks apoptotic cell death as induced by either a host-selective toxin or any of several necrotrophic pathogens, leading to protection against the disease caused by these pathogens. *p35* transgenic tobacco, passion-fruit, and narrow-leafed lupin all have increased tolerance to necrotrophic fungal pathogens and herbicides [21], [22], [23]. As noted above, a unifying aspect of these results is the fact that the plants with expression of anti-apoptotic genes are all resistant to necrotrophic pathogens, which kill plant cells and use the plant as a nutrient source for growth, colonization, and reproduction. Therefore, the inhibition of apoptotic pathways is a potential strategy for engineering necrotrophic disease resistance in plants.

The mechanism of action of these anti-apoptotic genes in plants, at present, is poorly understood. In this paper, the baculovirus anti-apoptotic protein P35, an IAP known to inhibit animal caspase, was effective in preventing PCD induced by VD-toxins. Although plants have no homologs of animal caspase genes and no identified caspase-dependent pathways, several identified proteases involved in plant PCD show caspase-like activities. The two serine proteases in *Avena sativa* exhibit caspase-like activity and have aspartate specificity, but contain a Ser-active site; they were involved in a PCD-induced protease cascade leading to the activation of another protease targeted to the chloroplast and cleaved Rubisco [46]. The vacuolar processing enzymes showed caspase-1 activity was essential for PCD induced by TMV [47] and fumonisin B1 [48], as well as for developmental cell death in the formation of the seed coat [49]. Chichkova et al. [50], [51] identified a novel PCD-related subtilisin-like protease that possessed caspase specificity and activity essential for PCD-related responses to TMV and abiotic stresses. Cell death-related proteins may exist that are functional equivalents with little or no sequence homology to animal counterparts, providing the functional domains to interact with the P35 and Op-IAP products.

*Verticillium dahliae*, which comprises a subset of necrotrophic pathogens, enters the plant by way of the root system and invades the protoxylem vessels. The molecular mechanisms of verticillium diseases are unclear. In this study, after VD-toxin treatment, cotton DNA was fragmented and formed characteristic ladders, and the percentage of dead protoplasts for the untransformed and transformed cotton all displayed an increase. These results were consistent with those of Wang et al. [9] who purified VdNEPs that induced DNA laddering of cotton suspension-cultured cells, and Palmer et al.[52]who purified an 18.5-kDa phytotoxic protein that induced more dead protoplasts of susceptible cotton cultivars than resistant cultivars. In addition, Chu et al. [40]reported that the secreted glycoproteins of *V. dahliae* play an important role in the wilting symptom and that native glycoproteins at concentrations higher than 5 mg/L resulted in cell death. Meyer et al. [7], [53]showed that cotton plasma membranes have high-affinity, specific binding sites for the protein lipopolysaccharide complex (PLPC) from *V. dahliae*, and that the PLPC induced symptoms of wilting and necrosis and inhibited the H^+^-ATPase activity of plasma membranes, stimulating phenylalanine ammonia-lyase activity. All this serves to illustrate that the secreted substances of *V. dahliae* are the key biochemical factors that cause the plant disease, which can trigger plant cells to die by induction of a PCD mechanism involved in pathogenesis.

Taken together, this work represents the first attempt to show heterologous expression of the baculovirus anti-apoptotic *p35* and *op-iap* genes in cotton plants. The results demonstrated that *p35* and *op-iap* gene expression partially protects cells from VD-toxin-induced PCD and enhances resistance to verticillium wilt disease. These results provide a potential strategy for engineering broad-spectrum disease resistance in plants and a good system for further study on plant PCD, as well as providing further evidence that pathogenesis-associated cell death in plants shares common regulatory and mechanistic features with PCD in animals.
